# DNA steganography: hiding undetectable secret messages within the single nucleotide polymorphisms of a genome and detecting mutation-induced errors

**DOI:** 10.1186/s12934-020-01387-0

**Published:** 2020-06-11

**Authors:** Dokyun Na

**Affiliations:** Department of Biomedical Engineering, School of Integrative Engineering, Chung-Ang University, 84 Heukseok-ro, Dongjak-gu, Seoul, 06974 Republic of Korea

**Keywords:** DNA encryption, Cell engineering, DNA barcode, Watermarking

## Abstract

**Background:**

As cell engineering technology advances, more complex synthetically designed cells and metabolically engineered cells are being developed. Engineered cells are important resources in industry. Similar to image watermarking, engineered cells should be watermarked for protection against improper use.

**Results:**

In this study, a DNA steganography methodology was developed to hide messages in variable regions (single nucleotide polymorphisms) of the genome to create hidden messages and thereby prevent from hacking. Additionally, to detect errors (mutations) within the encrypted messages, a block sum check algorithm was employed, similar to that used in network data transmission to detect noise-induced information changes.

**Conclusions:**

This DNA steganography methodology could be used to hide secret messages in a genome and detect errors within the encrypted messages. This approach is expected to be useful for tracking cells and protecting biological assets (e.g., engineered cells).

## Background

As synthetic biology and metabolic engineering technologies advance, industrially important engineered cells are being developed; these cells are considered as biological assets that should be protected [1–3]. Therefore, researchers have begun to develop methods to “watermark” cells. Conventional DNA watermarking methods involve the encryption of messages in the form of DNA sequences, which are then inserted into the genome, e.g., as DNA barcodes, or which are mixed with unrelated DNA fragments to hide the messages [4–7]. Decryption is simply carried out by polymerase chain reaction (PCR) or electrophoresis.

DNA sequences have attracted much interest as pieces of quaternary digit information that can be used to store information [8], solve problems [9–11], and encrypt messages [4–7]. DNA cryptography, i.e., the encryption of messages using DNA, has been used to cipher secret messages. Clelland et al. developed a method to hide encrypted messages [4]. A message is converted to a quaternary digit string and then replaced with a corresponding nucleotide sequence. This sequence, flanked by specific primer binding sites at both ends, is mixed with the fragmented human genome. The human genome provides background noise and allows the secret sequence to be concealed. To read the message, the specific primer set is required for PCR and sequencing. However, with currently available NGS technology, secret messages hidden using this approach can be easily found and such method cannot be applied to hide information in a genome. There was a report to make a watermark to track pathogens before distribution [12]. Pathogens could be used for bioterror or may be leaked from laboratories. In order to track and monitor the pathogens, DNA watermark using polymorphic regions was suggested. Briefly, the method introduces random mutations into a pathogen genome and then identifies pathogens that do not show significant phenotypic changes. Then, it could be assumed that the mutations were introduced into the polymorphic region and the mutated sequence can be used as a watermark. This method is interesting, because the watermark can be hidden in the polymorphic regions. However, this method requires random mutations and selection of genomes showing no phenotypic changes, which cannot store intended information and require laborious and time-consuming experiments. Thus, new methods are needed to better hide messages in DNA.

Accordingly, in this study, a new DNA steganography methodology was proposed to hide secret messages in variable regions [single nucleotide morphisms (SNPs)] of a genome. Through this method, a message was encrypted into a DNA sequence similar to other DNA cryptography methodologies [4]. Then the encrypted nucleotide sequence was inserted into the SNP regions of a genome. Because SNPs are naturally polymorphic, it becomes difficult to determine whether a nucleotide is an SNP or a part of an encrypted message. To overcome the limitation of DNA as an information storage module owing to the presence of mutations, a block sum check algorithm was employed to detect noise-induced information changes in network data transmission [13]. Using this algorithm, mutational errors could be easily detected and fixed, allowing the message to be stored for a long time. Overall, the DNA steganography methodology developed in this study (hiding messages in SNPs and using the block sum check algorithm to detect errors) could be useful for marking cells for management purposes and for protecting engineered cells.

## Results

### Identification of SNPs and SNP hotspots

**Fig. 1:**
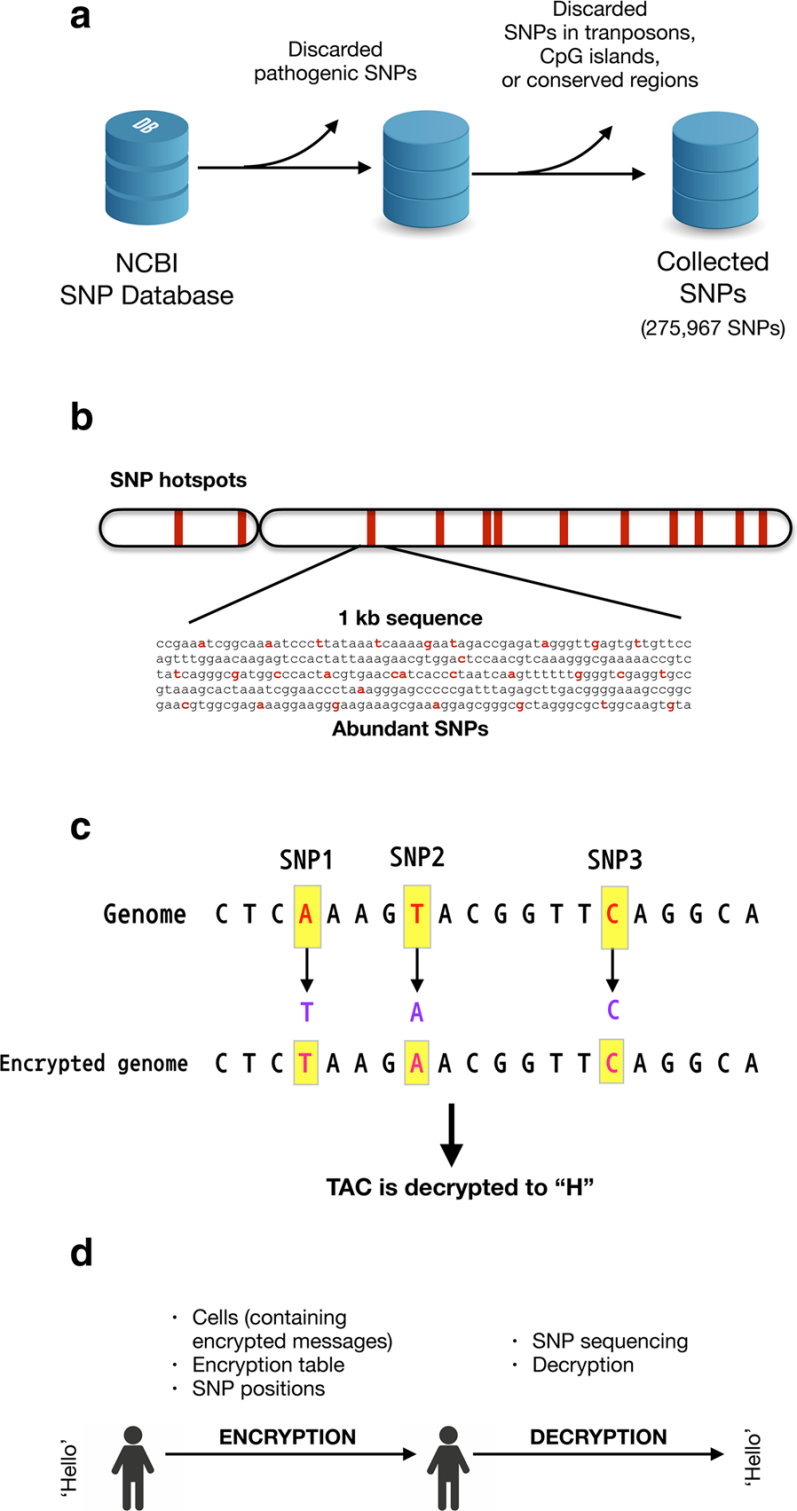
Collection of SNP data and exemplary encryption of the “H” character. **a** SNP dataset collection. SNPs were downloaded from NCBI dbSNP (ftp://ftp.ncbi.nih.gov/snp/latest_release/JSON). To use SNPs to store encrypted messages, pathogenic SNPs were discarded, and SNPs that had maximum variation (A/T/G/C) were then selected. In addition, SNPs that were in transposable elements, CpG islands, or conserved regions were discarded. Then, the sequences around SNPs (− 10 ~ + 10 nt, 21 nt in total) that were unique in the genomic sequences were selected. Through this process, 275,967 SNPs were found to be available for DNA steganography. **b** SNP hotspots. Regions that contained more than 35 SNPs within a 1 kb region were selected and named as SNP hotspots. The largest SNP number in a hotspot was 120 (Table 2). The SNPs in the hotspots were used to reduce the number of experiments. **c** An example of DNA steganography. The character “H” was encrypted into a DNA sequence according to the encryption table (Table 3), and each encrypted nucleotide replaced the original nucleotide at the predefined SNP positions. To construct a DNA sequence to decrypt, the SNP positions used for DNA insertion should be delivered to a recipient in addition to the encryption table. **d** Overall scheme of the secret message delivery and decryption

Firstly, dbSNP (build 153) was downloaded from NCBI to identify polymorphic regions within the human genome that could be used for hiding encrypted text. Unlike other organisms, many SNPs have been discovered in humans, providing sufficient information for DNA steganography. To this end, I searched for SNPs, allowing for one of four nucleotides (A/T/G/C) to be present at the position, and I discarded SNPs that were pathogenic (Table 1, Fig. 1a). Then, the sequences around the SNPs (21 nt-long sequence around SNPs) that were unique in the human genome were selected. Furthermore, the SNPs that were within transposable elements, CpG island, or conserved regions were discarded. For the identification of SNPs in transposable elements, the database of transposable elements (Dfam) [14] was used. For CpG island identification, the Sequence Manipulation Suite [15] was used to predict CpG island regions. For the identification of SNPs in conserved regions that may modify phenotypes, the conservation scores calculated by PhastCons [16] were used. The final number of selected SNPs was 275,967 (Table 1).

**Table 1:** SNPs in *Homo sapiens* that can be used for DNA steganography

Chr	SNPs	Chr	SNPs	Chr	SNPs	Chr	SNPs
1	19,986	2	23,308	3	18,488	4	17,537
5	16,424	6	15,276	7	15,544	8	19,950
9	13,249	10	13,388	11	13,782	12	11,837
13	8272	14	8539	15	8141	16	14,475
17	8099	18	7317	19	7131	20	6628
21	3882	22	4715

Theoretically, all SNPs can be used to store encrypted messages. However, current genome editing technologies, including CRISPR/Cas, are not capable of multiple genome editing simultaneously. For example, to encrypt a DNA sequence of 70 nucleotides, that requires 70 SNPs, 70 nucleotide-editing should be carried out. Thus, SNP hotspots were searched to facilitate genome modification. In this search, regions were selected that had more than 35 SNPs within a 1 kb region. If two hotspots are available, insertion of the encrypted sequence is possible just by two iterations of genetic recombination. There were five SNP hotspots having at least 35 SNPs within 1 kb. The largest hotspot contained 120 SNPs within a 1 kb region (chromosome 12, positions 88860531–88861530). For example, using this hotspot, the encrypted DNA sequence (70 nt) could be hidden through a one-step homologous recombination experiment. The found hotspots are listed in Table 2.

**Table 2:** SNP hotspots

Chr	Positions	SNPs
2	88860531–88861530	120
6	32663318–32664317	45
14	105863177–105864176	102
14	105864182–105865181	47
22	22880555–22881554	47

The selected SNPs were non-pathogenic. However, silent SNPs in coding sequences such as the third nucleotide of *CUN* encoding for leucine can be used to reduce phenotypic changes more. However, such restriction dramatically reduces the space for information storage from 275,967 nt to 8790 nt and makes it difficult to find SNP hotspots. No hotspots for DNA steganography were found when the 8790 nt were used. Therefore, multiple genome editing is inevitable for the introduction of encrypted messages into the codon degeneracy positions and thereby it also makes the DNA steganography less applicable.

### Encryption of plain text into DNA sequence

Next, plain text was encrypted using a substitution cipher [17] (Table 3). There are many other encryption algorithms, including Data Encryption Standard (DES) [18], Advanced Encryption Standard (AES) [19], and Rivest-Shamir-Adleman (RSA) [20]. These algorithms could be also used instead of the simple substitution method. In this study, for simplicity and proof-of-concept of DNA steganography, a substitution method was used.

**Table 3:** DNA encryption table

	First nucleotide			
	A	T	G	C
Second nucleotide				
A	AAA (A)	TAA (E)	GAA (I)	CAA (M)
	AAT (B)	TAT (F)	GAT (J)	CAT (N)
	AAG (C)	TAG (G)	GAG (K)	CAG (O)
	AAC (D)	TAC (H)	GAC (L)	CAC (P)
T	ATA (Q)	TTA (U)	GTA (Y)	CTA (c)
	ATT (R)	TTT (V)	GTT (Z)	CTT (d)
	ATG (S)	TTG (W)	GTG (a)	CTG (e)
	ATC (T)	TTC (X)	GTC (b)	CTA (f)
G	AGA (g)	TGA (k)	GGA (o)	CGA (s)
	AGT (h)	TGT (l)	GGT (p)	CGT (t)
	AGG (i)	TGG (m)	GGG (q)	CGG (u)
	AGC (j)	TGC (n)	GGC (r)	CGC (v)
C	ACA (w)	TCA (1)	GCA (5)	CCA (9)
	ACT (x)	TCT (2)	GCT (6)	CCT (0)
	ACG (y)	TCG (3)	GCG (7)	CCG ()
	ACC (z)	TCC (4)	GCC (8)	CCC (.)

**Fig. 2:**
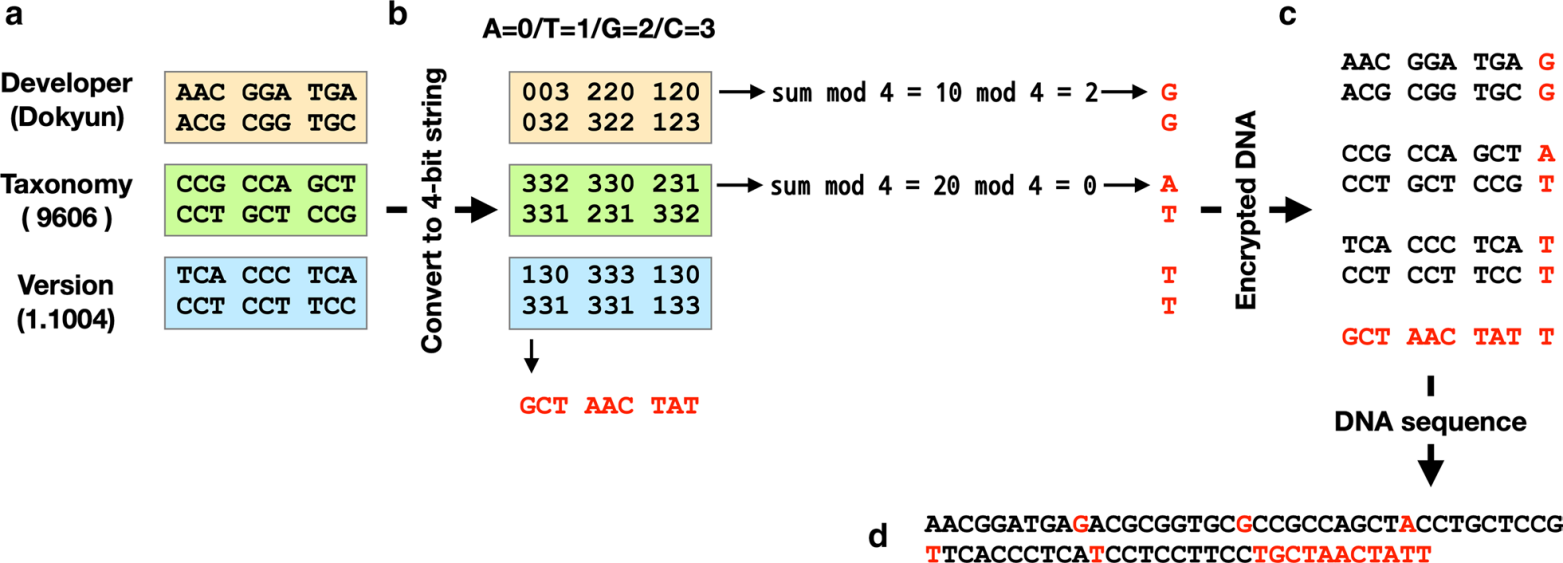
Block sum check algorithm of the encrypted DNA sequence. **a** Three information types (developer, taxonomy, and version) were encrypted from plain text into the DNA sequence according to the encryption table (Table 3). “9606” denotes *Homo sapiens*. The nucleotide sequence was then arranged in 2D. **b** For block sum check, nucleotides were changed to quaternary digit numbers (A = 0, T = 1, G = 2, C = 3). For each row, the sum of the numbers was divided by 4, and the remainder was then converted to a nucleotide. For example, the sum of the first row was divided by 4, and its remainder was 2. This 2 was then converted to a corresponding nucleotide, G. For each column, the same process was iterated to obtain parity nucleotides. **c** The parity nucleotides for error check are shown in red color. **d** The final 1D sequence of encrypted and mutation-detectable sequence was constructed

As shown in Fig. 1c, a character is converted to a DNA triplet using keys similar to a codon table (Table 3). The character H was encrypted using the encryption table to TAC, and this TAC sequence could be inserted into predefined SNP positions to hide the encrypted character. For example, “Hello” can be replaced with the DNA sequence TAC CTG TGT TGT GGA. “Dokyun” in Fig. 2 is encrypted as AAC GGA TGA ACG CGG TGC.

### Block sum check to detect mutations

Next, to detect mutations in the encrypted DNA sequence, a block sum check method was employed, as is commonly used in network data transmission [13]. As shown in Fig. 2a, first, the words “Dokyun,” “9606” and “1.1004” were encrypted into DNA sequences, and the sequences were arranged in 2D. To check the integrity of the sequence, additional nucleotides were attached to each row and column. For example, the sequence in the first row (AACGGATGA) was converted to a quaternary digit string (003220120 where A = 0/T = 1/G = 2/C = 3). Then, the sum of the numbers (0 + 0 + 3 + 2 + 2 + 0 + 1 + 2 + 0) was divided by 4, and the remainder 2 was converted to the nucleotide G. This process was iterated until the last row, and the additional nucleotides (G, G, A, T, T, T) were added to each row (Fig. 2b). The same calculation was iterated for each column. For example, the sum of the first column (0 + 0 + 3 + 3 + 1 + 3) was divided by 4, and the remainder 2 was converted to a nucleotide G. The complete additional nucleotides (parity nucleotides) are shown in red in Fig. 2c and d.

The additional nucleotides were used for checking the integrity of the encrypted DNA sequence and for detecting errors caused by mutations. For example, if the first nucleotide A was mutated to T, the remainder of the first-row sum divided by 4 was 3, corresponding to C; this did not match G in the parity nucleotide. Using this approach, the mutation in the first row and first column can be detected.

### Decryption from DNA sequence

To decrypt the secret message hidden in the genome, a user has to know the encryption table and the positions of the SNPs used. The decryption was the reverse of the process depicted in Fig. 2. First, if a message was hidden within an SNP hotspot, the region could be easily sequenced because the hotspot was only 1 kb long. Then, the nucleotides in the predefined SNP positions were combined to generate a 1D DNA sequence. Second, the DNA sequence was rearranged in 2D, and each row contained 10 nucleotides (9 for the message and 1 for the error check). The additional parity nucleotides were used to determine whether there were mutational changes. Third, if there were no mutations, the sequence in the main body (black in Fig. 2c) was rearranged in a 1D sequence. Then, similar to mRNA translation, DNA triplets were translated into characters using the encryption table. After this process, the decrypted message could be obtained.

**Fig. 3:**
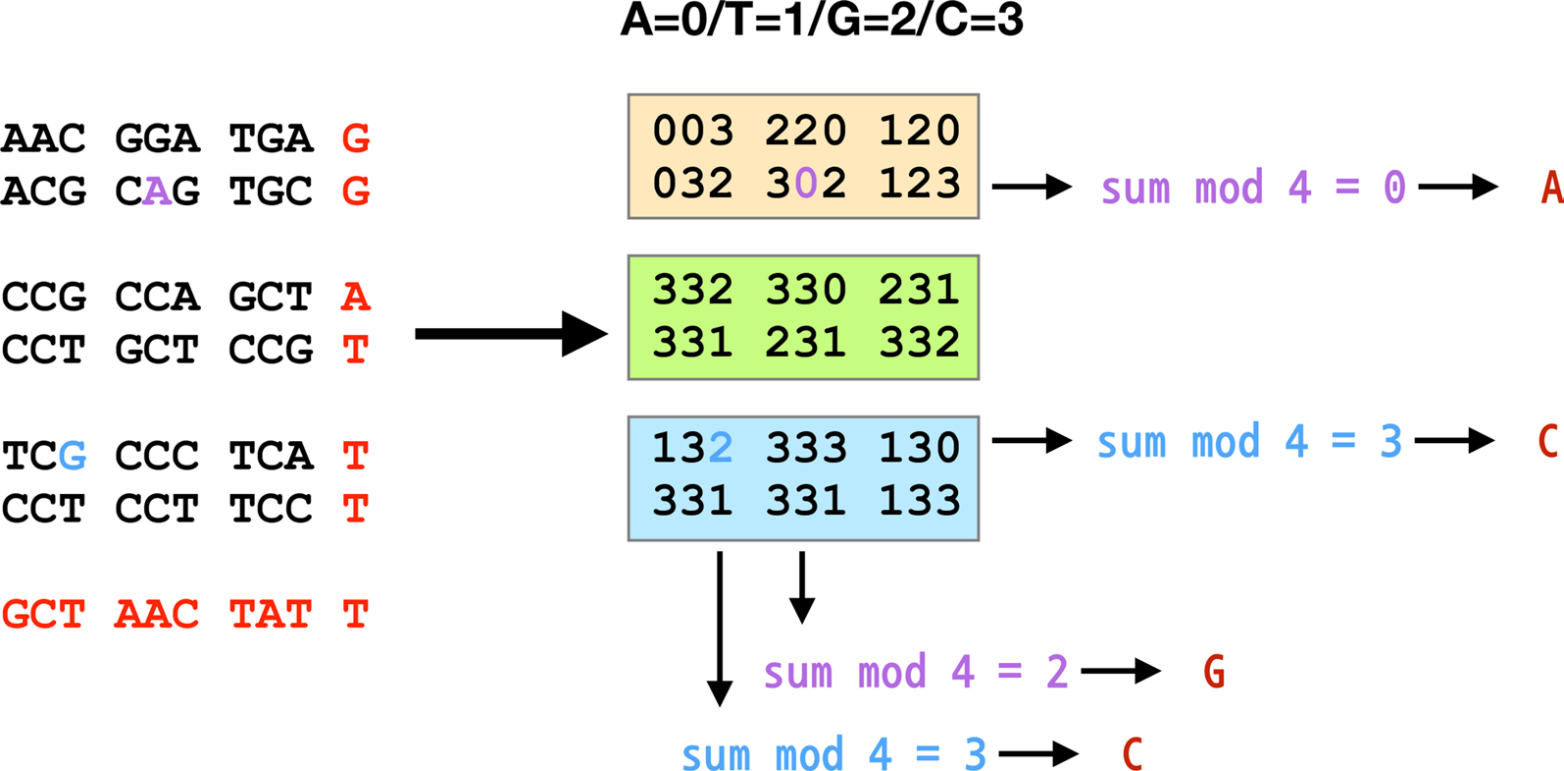
Mutation detection and deduction of the original nucleotide. In this encrypted DNA message, two mutations were introduced (G→A colored in violet and A→G colored in cyan). The mutations were detected by calculating the remainders and comparing with the parity nucleotides. Deduction of the original nucleotide can be performed by finding a number (nucleotide) that satisfies the row and column parity numbers (nucleotides)

When mutations were introduced within the encrypted message, they can be easily detected and the original nucleotides can be deduced. An example is shown in Fig. 3. Two mutations were introduced into the message (G→A colored in violet and A→G colored in cyan). Based on the block-sum-check algorithm, the parity nucleotide of the second row must be G. However, the remainder when divided by 4 is 0 that denotes A. This mismatch allows to know that a mutation was introduced in the second row. Likewise, the remainder of the fifth column when divided by 4 is 3 denoting C. However, the parity nucleotide must be A. Consequently, it can be found that the A is a mutated nucleotide. To deduce the original nucleotide, the nucleotide should satisfy the parity nucleotides (row and column). For the second row, the remainder must be 2 because the parity nucleotide is G. In addition, for the fifth column, the remainder must be 0 because the parity nucleotide is A. Therefore, the number (nucleotide) that satisfies the two conditions is 2 (G). Consequently, it can be deduced that the A was mutated from G. Likewise, the A→G mutation (cyan) can be deduced through the same process.

### SNP distributions in other species

**Fig. 4:**
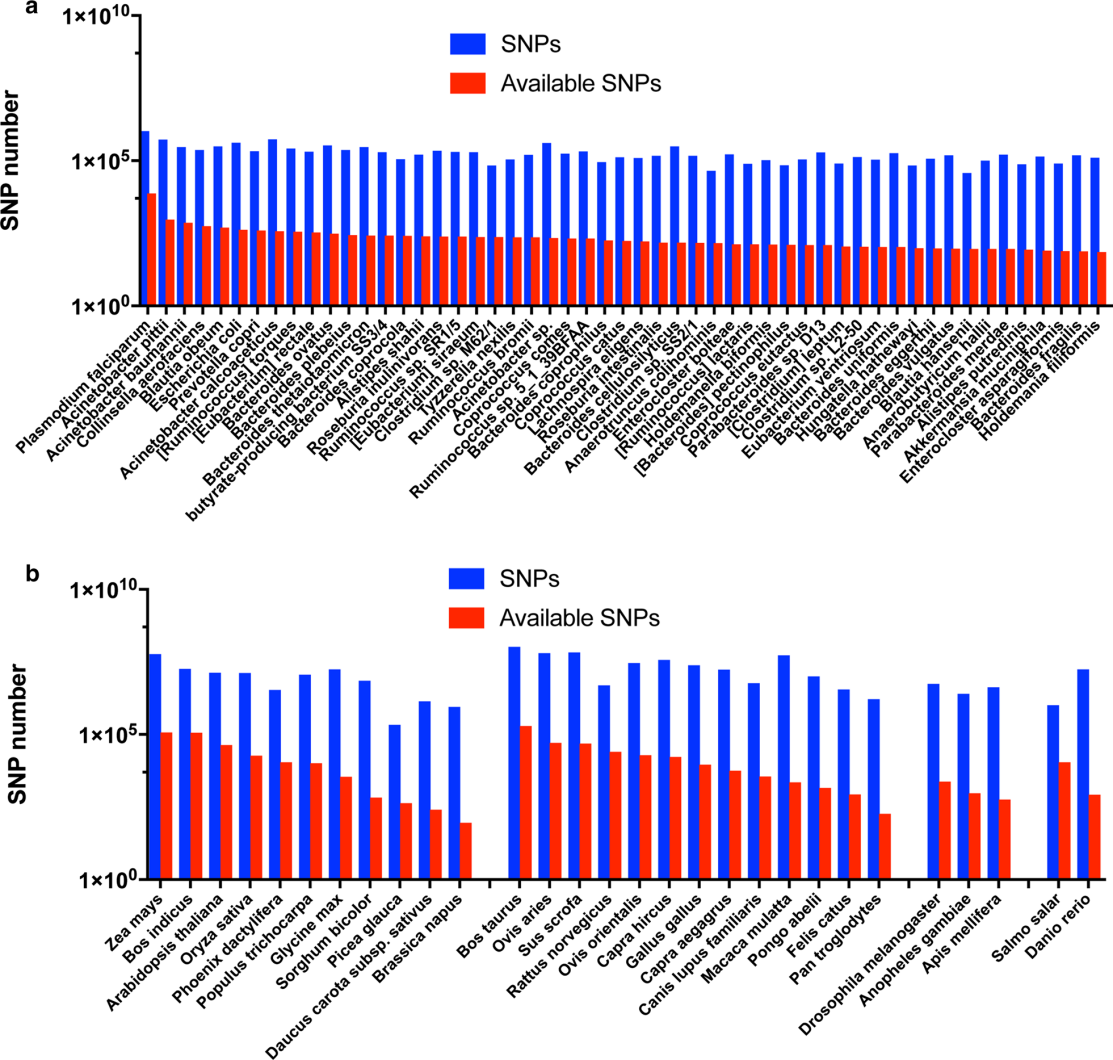
SNP distributions across species. SNP datasets of other species were obtained from dbSNP, and their distributions of total SNPs (blue) and available SNPs (red, SNPs that can be any of 4 nucleotides (A/T/G/C) and thereby can be used for DNA steganography) were analyzed. The SNP numbers in bacteria species (**a**) and other species (plants, mammals, insects, and fish) (**b**) are shown

The DNA steganography was proved its usefulness using human SNPs in this study. For practical applications, the SNPs should be available in other species as well. Therefore, the SNP datasets of 311 species were obtained from dbSNP and the species that have fewer than 70 SNPs (SNPs that can be any of four nucleotides (A/T/G/C)) were discarded. As a result, I obtained 53 bacteria species, 11 plant species, 13 mammalian species, two insect species, and two fish species (Fig. 4a, b). This represents that SNPs are widely available in many species even in bacteria, and the DNA steganography would be possibly applied to any species that have SNPs.

## Discussion

The key feature of our developed DNA steganography method is hiding messages in natural SNPs. Humans have many SNPs and SNP hotspots. However, this approach cannot be applied to well-established model organisms whose DNA sequences are already determined and who have only a few SNPs. However, any other species with a sufficient number of SNPs can be used as a carrier of secret messages. For example, 70 SNPs were enough to hide the information in Fig. 2a. As the message length increases, the required number of SNPs also increases.

One of the potential applications of DNA steganography is DNA barcoding. In general, DNA barcodes are embedded into the genome; thus, the barcode may be affected by mutations. Because the DNA steganography method developed in this study employed an error checking algorithm using block sum check, the DNA steganography approach could be used as a new DNA barcoding system.

Another potential application of this approach is to “watermark” engineered cells to indicate that the cells are from a specific company or researcher. Thus, DNA steganography can be used to protect the intellectual property of engineered cells. As the technologies of synthetic biology and metabolic engineering advance, engineered cells are constantly being developed. In the bioindustry, it is necessary to encrypt ownership information within the genome of cells. Because the information will be hidden in variable SNP regions, the hidden message cannot be detected by conventional technologies, such as NGS. In addition, the method can detect errors, and mutations in the hidden message can then be easily detected. Therefore, the proposed DNA steganography method may be a feasible approach for protecting engineered cells.

## Conclusions

In summary, in this study, a DNA steganography methodology was proposed to encrypt secret messages in DNA sequences and hide the messages in SNPs to prevent from detection. The advantages shown by the method were as follows: (1) the encrypted message could not be detected using conventional experimental technologies, and (2) the message was mutation tolerant, allowing errors to be easily detected and fixed if possible. The DNA steganography method can theoretically use any SNPs to hide messages, but in reality, only a few SNP hotspots are available to use because of current genome editing techniques. As multiplex genome editing techniques advance, the DNA steganography can use all SNPs to hide messages and which makes it more difficult to be hacked.

As cell-engineering technology advances and different types of engineered cells are being developed, intellectual property issues are expected to arise. Thus, the DNA steganography approach developed in this study may be a feasible method to protect engineered cells by “watermarking.”

## Methods

### Encryption of information to DNA sequence

For the encryption of plain text into a DNA sequence, a substitution cipher was used (Table 3) as a proof-of-concept of the DNA steganography methodology. A DNA triplet like a codon corresponds to a character or number. Therefore, text can be translated to a DNA sequence. For example, ‘Hello’ is converted to a sequence of ‘TAC CTG TGT TGT GGA ‘.

### Block sum check to detect mutations

One of the drawbacks of saving information into DNA sequences is its mutational change. Mutational change of a nucleotide may change the meaning of an encrypted message. For example, ‘CCA TCA TCA’ corresponds to ‘911’. A mutational change of the first nucleotide, C, to T (‘**T**CA TCA TCA’) is now translated to ‘111’. To find mutations, a block sum check algorithm was employed, which is used to detect errors in network data transmission.

**Fig. 5:**
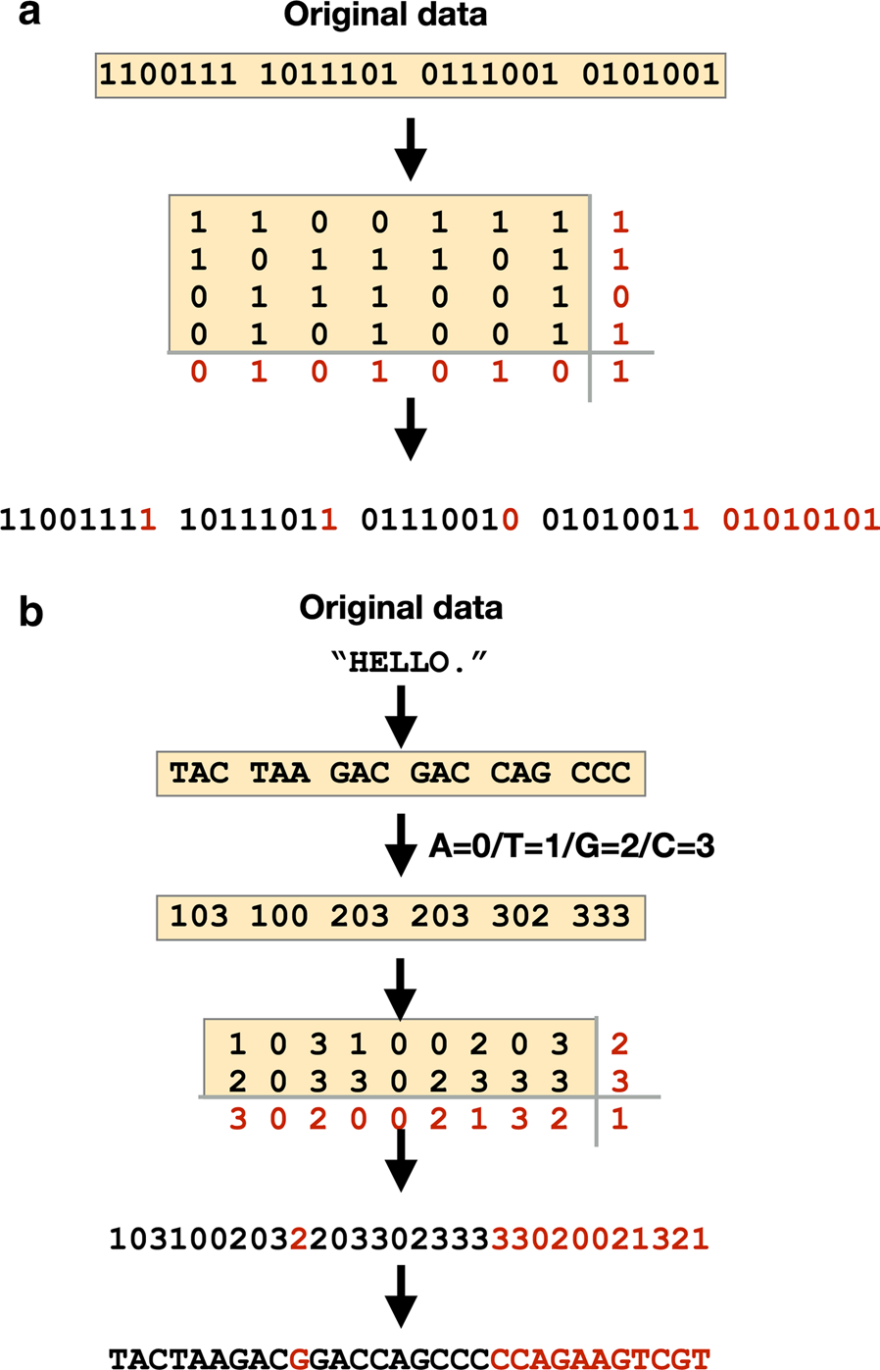
Block sum check algorithm to detect errors. **a** Conventional block sum check in data transmission. **b** Block sum check for quaternary digit data (DNA)

The first step of conventional block sum check is to divide data. As shown in Fig. 5a, the bit string was divided into 7-bit strings. The data is arranged in 2D, and then parity bits are added to each row and each column. For example, in Fig. 5a, the numbers in the first row is summed and then divided by 2. The remainder, 1, is added to the end of the first row. This ‘1’ is an additional bit (parity bit). Likewise, the sum of first column is divided by 2 and the remainder, ‘0’, is added to the end of the first column. The added parity bits are shown in red in Fig. 5a. The last step is to arrange the data in 1D.

In the DNA steganography methodology, the same block sum check algorithm was applied, but the only difference is that DNA is quaternary digit. As shown in Fig. 5b, a text “HELLO.” is encrypted into ‘TAC TAA GAC GAC CAG CCC” according to the encryption table (Table 3). This DNA sequence is converted to quaternary digit. The numbers are arranged in 2D and sums of row and column are divided by 4, and finally parity numbers are added to each row and column. The modified encrypted sequences are then ‘TACTAAGAC**G**GACCAGCCC**CCAGAAGTCGT**’.

If there is a mutation in the sequence, the remainders of row/column would be different from its parity numbers. Thus, by calculating the numbers (nucleotides), errors can be detected and fixed if possible.

### Decryption from DNA sequence

Decryption is the reverse process of the block sum check and encryption. An encrypted DNA sequence is converted to quaternary digit, and parity numbers are checked. If there are no errors in the sequence, the nucleotides except parity numbers are translated by the encryption table.

### Identification of SNP hotspots

To collect SNPs, dbSNP was downloaded from NCBI (build 153). Since SNPs are naturally polymorphic, SNPs can be A/T, A/G, C/A/G, A/T/G/C, etc. To store encrypted DNA sequences, SNPs that can be any of nucleotides (A/T/G/C) were collected, but the frequencies of the nucleotides were not considered. To avoid diseases or cell death, pathogenic SNPs were then discarded (Fig. 2a) by selecting only benign SNPs or the SNPs that did not have a particular description. In addition, the SNPs, that were redundant in the human genome or that exist within transposable elements, CpG islands, or conserved regions, were discarded. For uniqueness check, the sequences − 10 ~ + 10 around SNPs (21 nt in total) were used to find unique sequences in the human genome. The 21 nt (4^21^ = 4.4 × 10^12^) was enough to avoid random matches. For transposable elements, the database Dfam that contained transposable element information was used [14]. For CpG island identification, the Sequence Manipulation Suite [15] was used to predict whether the sequences around SNPs (− 100 nt ~ + 100 nt, 201 nt in total) were CpG island regions or not. Since SNPs may be involved in conserved regions in which the SNPs may alter the function of genes or change phenotypes, such SNPs were also discarded using the conservation scores calculated by PhastCons with a threshold of 0.6. The number of remaining SNPs were 275,967 (Table 1).

Current genome editing technologies are not able to modify nucleotides at multiple positions. For convenient storage of encrypted DNA sequences into SNPs, SNP hotspots were identified (Fig. 1b and Table 2). In this study, a hotspot is defined as a 1 kb-long region that include more than 35 SNPs. The SNP hotspots are shown in Table 2.

### SNPs in other species

SNP datasets of other species were also download from dbSNP (https://ftp.ncbi.nih.gov/snp/organisms/archive/). The SNP datasets of 311 different species were obtained. The species that have fewer than 70 SNPs that can be any of four nucleotides (A/T/G/C) were discarded. As a result, I obtained 53 bacteria species, 11 plant species, 13 mammalian species, two insect species, and two fish species.

## Data Availability

Not applicable.
